# Supplementary material for: Positive skill transfer in balance and speed control from balance bike to pedal bike in adults: A multiphase intervention study
Source: PLoS One. 2024 Feb 29;19(2):e0298142. doi: 10.1371/journal.pone.0298142 (PMC10903920; doi:10.1371/journal.pone.0298142)
Supplement: S1 Appendix — (DOCX) [file pone.0298142.s001.docx]

**S1 Appendix**

STROBE Statement—checklist of items that should be included in reports of observational studies.

|  | Item No. | Recommendation | Page  No. | Relevant text from manuscript |
| --- | --- | --- | --- | --- |
| **Title and abstract** | 1 | (*a*) Indicate the study’s design with a commonly used term in the title or the abstract | 2 – 3 | “…Methods: In Phase 1, a case-control observational study was conducted in which 13 cyclists and 8 non-cyclists completed balance bike tests…” |
|  |  | (*b*) Provide in the abstract an informative and balanced summary of what was done and what was found | 2 – 3 | “…The results in Phase 1 showed that cyclists performed better on the balance bike than non-cyclists …” |
| Introduction | | | |  |
| Background/rationale | 2 | Explain the scientific background and rationale for the investigation being reported | 4 – 7 | “Cycling is …” |
| Objectives | 3 | State specific objectives, including any prespecified hypotheses | 6 – 7 | “…this study adopted … single-arm trial” |
| Methods | | | |  |
| Study design | 4 | Present key elements of study design early in the paper | 7 | “This study was a multi-phase intervention study including a case-control observational study in Phase 1 and an 8 × 20-minute balance bike training single-arm trial in Phase 2 (Fig. 1). |
| Setting | 5 | Describe the setting, locations, and relevant dates, including periods of recruitment, exposure, follow-up, and data collection | 7  7 – 8 | “From June 2021 to August 2022, healthy adults were recruited on the basis of cycling experience (with or without) by convenience sampling (via online and poster advertising).  “Participants  In Phase 1 …” |
| Participants | 6 | (*a*) *Cohort study*—Give the eligibility criteria, and the sources and methods of selection of participants. Describe methods of follow-up  *Case-control study*—Give the eligibility criteria, and the sources and methods of case ascertainment and control selection. Give the rationale for the choice of cases and controls  *Cross-sectional study*—Give the eligibility criteria, and the sources and methods of selection of participants | 7 | *Case-control study*—  “… healthy adults were recruited on the basis of cycling experience (with or without) by convenience sampling (via online and poster advertising). The inclusion criterion is (1) … The eligibility criteria were the same in Phases 1 and 2.” |
|  |  | (*b*) *Cohort study*—For matched studies, give matching criteria and number of exposed and unexposed  *Case-control study*—For matched studies, give matching criteria and the number of controls per case | 8 | “…participants were classified into two groups: cyclist group and non-cyclist group. Studies typically identified three criteria for independent cycling: the ability to self-launch, riding a bike continuously for a certain distance, and braking without assistance [6]. However, the combination of criteria varied across studies. Since braking using the handlebar is not part of a balance bike’s design, participants in this study who were able to self-launch and ride a pedal bike for 5 minutes continuously were classified as the cyclist group, while those who were unable to do that were classified as the non-cyclist group.” |
| Variables | 7 | Clearly define all outcomes, exposures, predictors, potential confounders, and effect modifiers. Give diagnostic criteria, if applicable | 8  10 – 12 | “…the times of completion for two assigned balance bike manoeuvres were recorded. The research team used the information collected in Phase 1 to develop an eight 20-minute balance bike training programme that attempted to address non-cyclists’ needs on acquiring riding skills on a balance bike.  “*Physical functional capacity …*  *Balance bike testing…*  *Cycling Confidence …*  *Self-perception of the learning experience …*  *Cycle Independently …*” |
| Data sources/ measurement | 8* | For each variable of interest, give sources of data and details of methods of assessment (measurement). Describe comparability of assessment methods if there is more than one group | 10 – 13 | *“Motor-skill engagement (MSE) on a balance bike…*  *Physical functional capacity …*  *Balance bike testing…*  *Cycling Confidence …*  *Self-perception of the learning experience…*  *Cycle Independently …*” |
| Bias | 9 | Describe any efforts to address potential sources of bias | 13 | “A fully Bayesian inferential statistical approach was used to provide probabilistic statements [18]. Analysis in Phase 1, due to the lack of strong a priori evidence, non-informative prior was used [19]. A Bayesian two-sample t-tests were used to compare the outcome variables between cyclists and non-cyclists. Then, new prior distributions of cyclists and non-cyclists on the outcome parameters were collected.” |
| Study size | 10 | Explain how the study size was arrived at | 14 | “This study adopted Bayesian updating with a simulation-based approach for sample size calculation in Phase 1, sample size was continued to increase until the Bayes factor was sufficient, indicating strong evidence in favour of the alternative hypothesis.” |

Continued on next page

| Quantitative variables | 11 | Explain how quantitative variables were handled in the analyses. If applicable, describe which groupings were chosen and why | 13  10  11  11  11 – 12  12 | “The distance travelled (km) by each participant on balance bike was collected using cycle computer (CC-RS200W, Cateye Quick Cyclocomputer, Osaka, Japan). Participants were able to read the data monitor attached to the handlebar. The data was also used to determine the MSE on a balance bike during the training programme in Phase 2.  “The average jump height of three trials was used for analysis.”  “The average time completion (s) of five trials were assessed by a pair of timing gates (Brower Timing Systems, Utah, USA) that were placed at the start and finish lines.”  “Participants were asked to rate their confidence to ride a pedal bike on a five-point Likert scale (1 = not confident; 2 = slightly confident; 3 = somewhat confident; 4 = fairly confident; 5 = very confident).  Before the pedal bike trial at T2, a structured interview was conducted to assess the participants’ perception regarding their acquisition of cycling skills during the programme. The interview lasted for 3 minutes and consisted of two questions: Q1) How did you feel after completing the balance bike training programme? and Q2) Which specific elements do you feel you developed after completing the balance bike training programme that contributed to changes of your cycling confidence?”  “In this study, being able to cycle independently was defined as when a participant can self-launch and ride a pedal bike for 5 minutes continuously. At the end of the balance bike training programme, participants were invited to ride a 20-inch conventional pedal bike, which was of the same size as the balance bike used in the training. The time taken by participants to cycle independently was recorded and reported, in 15-minute intervals: below 15 minutes, 16 to 30 minutes, 30 to 45 minutes, and 45 to 60 minutes. The trial had a maximum time limit of 60 minutes.” |
| --- | --- | --- | --- | --- |
| Statistical methods | 12 | (*a*) Describe all statistical methods, including those used to control for confounding | 13 – 14 | “The SPSS Statistics 28.0 … large effect sizes, respectively” |
|  |  | (*b*) Describe any methods used to examine subgroups and interactions | Not applicable |  |
|  |  | (*c*) Explain how missing data were addressed | Not applicable |  |
|  |  | (*d*) *Cohort study*—If applicable, explain how loss to follow-up was addressed  *Case-control study*—If applicable, explain how matching of cases and controls was addressed  *Cross-sectional study*—If applicable, describe analytical methods taking account of sampling strategy | Not applicable |  |
|  |  | (*e*) Describe any sensitivity analyses | 13 – 14 | “…A fully Bayesian inferential statistical approach was used to provide probabilistic statements [18]. Analysis in Phase 1, due to the lack of strong a priori evidence, non-informative prior was used [19]. A Bayesian two-sample t-tests were used to compare the outcome variables between cyclists and non-cyclists. Then, new prior distributions of cyclists and non-cyclists on the outcome parameters were collected.” |
| Results | | | | |
| Participants | 13* | (a) Report numbers of individuals at each stage of study—eg numbers potentially eligible, examined for eligibility, confirmed eligible, included in the study, completing follow-up, and analysed | 8 | “A total of 21 participants took part in Phase 1…  “In Phase 2, another 12 non-cyclists were recruited using the same criteria to…” |
|  |  | (b) Give reasons for non-participation at each stage | 8 – 9 | “In Phase 2 … One individual withdrew from the study due to personal reasons.” |
|  |  | (c) Consider use of a flow diagram | 7 | “This study was a multi-phase intervention study including a case-control observational study in Phase 1 and an 8 × 20-minute balance bike training single-arm trial in Phase 2 (Fig. 1).” |
| Descriptive data | 14* | (a) Give characteristics of study participants (eg demographic, clinical, social) and information on exposures and potential confounders | 8 – 9 | “A total of 21 participants took part in Phase 1…  “In Phase 2, another 12 non-cyclists were recruited using the same criteria to…” |
|  |  | (b) Indicate number of participants with missing data for each variable of interest | Not Applicable |  |
|  |  | (c) *Cohort study*—Summarise follow-up time (eg, average and total amount) | Not Applicable |  |
| Outcome data | 15* | *Cohort study*—Report numbers of outcome events or summary measures over time | Not Applicable |  |
|  |  | *Case-control study—*Report numbers in each exposure category, or summary measures of exposure | 15 | [Table 2] |
|  |  | *Cross-sectional study—*Report numbers of outcome events or summary measures | Not Applicable |  |
| Main results | 16 | (*a*) Give unadjusted estimates and, if applicable, confounder-adjusted estimates and their precision (eg, 95% confidence interval). Make clear which confounders were adjusted for and why they were included | 14 – 20 | “Cyclists demonstrated …  “…Out of the eleven participants, eight were able to self-launch and ride a pedal bike continuously within 15 minutes, while the remaining three were able to cycle independently within 16 to 30 minutes (n = 1) and 45 to 60 minutes (n = 2).” |
|  |  | (*b*) Report category boundaries when continuous variables were categorized | Not Applicable |  |
|  |  | (*c*) If relevant, consider translating estimates of relative risk into absolute risk for a meaningful time period | Not Applicable |  |

Continued on next page

| Other analyses | 17 | Report other analyses done—eg analyses of subgroups and interactions, and sensitivity analyses | Not Applicable |  |
| --- | --- | --- | --- | --- |
| Discussion | | | | |
| Key results | 18 | Summarise key results with reference to study objectives | 21 | “In this multi-phase intervention study, we first examined … for every adult to learn how to cycle with dignity.” |
| Limitations | 19 | Discuss limitations of the study, taking into account sources of potential bias or imprecision. Discuss both direction and magnitude of any potential bias | 27 – 28 | “Our study had several limitations that…” |
| Interpretation | 20 | Give a cautious overall interpretation of results considering objectives, limitations, multiplicity of analyses, results from similar studies, and other relevant evidence | 27 – 28 | “The balance bike manoeuvres … more diverse population and make the findings more generalizable.” |
| Generalisability | 21 | Discuss the generalisability (external validity) of the study results | 28 | “Future studies should measure and control physical activity levels to include a more diverse population and make the findings more generalizable.” |
| Other information | |  | | |
| Funding | 22 | Give the source of funding and the role of the funders for the present study and, if applicable, for the original study on which the present article is based | Not Applicable | Stated in a separate “Financial Disclosure Statement” |

*Give information separately for cases and controls in case-control studies and, if applicable, for exposed and unexposed groups in cohort and cross-sectional studies.

**Note:** An Explanation and Elaboration article discusses each checklist item and gives methodological background and published examples of transparent reporting. The STROBE checklist is best used in conjunction with this article (freely available on the Web sites of PLoS Medicine at http://www.plosmedicine.org/, Annals of Internal Medicine at http://www.annals.org/, and Epidemiology at http://www.epidem.com/). Information on the STROBE Initiative is available at www.strobe-statement.org.
