# Supplementary material for: Positive skill transfer in balance and speed control from balance bike to pedal bike in adults: A multiphase intervention study
Source: PLoS One. 2024 Feb 29;19(2):e0298142. doi: 10.1371/journal.pone.0298142 (PMC10903920; doi:10.1371/journal.pone.0298142)
Supplement: Attachment — Submitted filename: PONE-D-23-13359_Point-by-Point-Response_231128.docx [file pone.0298142.s004.docx]

Journal:	PLOS ONE Decision: Revision required [PONE-D-23-13359R1]

Manuscript Status:	Minor revision

Manuscript ID:	**PONE-D-23-13359R1**

Type:	Article

**Positive Skill Transfer in Balance and Speed Control from Balance Bike to Pedal Bike in Adults: A Multiphase Intervention Study**

**Point-by-Point Response**

# Reviewer 1

| Point raised by Reviewer #1 | **Response by author** |
| --- | --- |
| The authors addressed many concerns adequately. However, some issues remain insufficiently addressed. Further proof reading is needed. | We made further proof reading and provided our best effort to address the concerns. Please find the point-to-point response below: |
| Abstract –  Avoid inflated language like “extreme” evidence, let the data speak for the impact. | The abstract has been updated according to the comment, we agree to “let the data speak the impact”.  Line 37 – 40 “…with Bayes factor analyses providing evidence of this difference, BF01 = 0.228 in the 15 m sprint test and BF01 = 0.138 in the two-turn curved sprint test. The novel training programme in Phase 2 remarkable effectiveness in improving their balance bike riding performance…”  Line 44 – 45 “…It provides extreme evidence that healthy adults can learn to ride independently with the help of a balance bike, a tool that's commonly used for teaching children…”  Besides, to clarify the interpretation of Bayes Factor, a paragraph in data analysis has been updated and a updated reference was added.  Page 14, line 286 – 292  “The strength of the evidence for the null hypothesis against the alternative hypothesis (or models) can be expressed as a Bayes Factor (BF01), which is an odds ratio. The size of BFs can be interpreted as providing anecdotal (BF01 = 1/3–1), moderate (BF01 = 1/10–1/3), strong (BF01 = 1/30–1/10), very strong (BF01 = 1/100–1/30), and extreme (BF01 < 1/100), in favour of alternative hypothesis compared to null hypothesis [2320, 21]. Evidence for the null hypothesis was set as anecdotal (BF01 = 1–3), moderate (BF01 = 3–10), strong (BF01 = 10–30), very strong (BF01 = 30–100), and extreme (BF01 > 100) [20, 21].”  New reference:  Kelter R. “Bayesian alternatives to null hypothesis significance testing in biomedical research: A non-technical introduction to Bayesian inference with JASP. BMC Med. Res. Methodol. 2020;20(1): 142, <https://doi.org/10.1186/s12874-020-00980-6>. |
| Introduction –  I suggest simplifying the background to be more directly related to your study purpose and outcomes.  For example, you provide information about parents teaching children to ride, however, this is not a purpose or outcome of your study, so it is not relevant.  Also, is there evidence to suggest that increased cycle track miles have results in earlier learning. I do not see evidence of a cause-and-effect relationship. | We agree to simplify the introduction.  Line 62 – 72  “…Learning to cycle independently is a common rite of passage for children… potentially delaying the age at which individuals learn to cycle independently [3].”  Therefore, the reference list is also updated.  Page 30 – 35 |
| Line 84, be more specific of what challenges balance bikes have helped to overcome. It does not seem like they overcome all the challenges listed before this statement.  Introduction can be examined to improve flow. | Thanks for the comments. We revised the paragraph and clarified what challenges were.  Line 73  “In recent times, balance bikes have emerged as a popular alternative to overcome the challenges associated with traditional learning approaches for cycling. These challenges primarily involve ineffective methods and negative experiences. Balance bikes, which lack pedals and allows rider to walk or sprint while seated [5], offer a more intuitive and effective way to acquire skills and improve cycling stability. In fact, studies have shown that balance bikes are more effective in helping riders learn the essential skill of maintaining balance on two wheels compared to bikes with training wheels, which prioritize on pedalling skills [3, 4]. With the right support, including access to affordable, appropriately sized adult balance bikes, along with effective learning design, individuals of all ages can overcome these barriers and learn to cycle independently.” |

# Reviewer 4

| **Point raised by Reviewer #4** | **Response by author** |
| --- | --- |
| I think that this study is original and that the results can contribute to practical application, and given results could be used for future research on the same topic | Thanks for the positive comments. |

# Reviewer 5

| **Point raised by Reviewer #3** | **Response by author** |
| --- | --- |
| very interesting study, and very current, considering that it includes elderly people. | Thanks for the positive comments. |
| A couple of suggestions:  - the tables should be formatted exactly the same way (table 1 and the same table)  - the title of the table should be (in one table it is bold, and in the other table it is not) | Table 1 and 2 had been updated.  Page 15, line 286  Page 16, line 297 – Page18, line 301 |
| - Hypotheses are mentioned in the Discussion (paragraph 381-385), but I did not notice that the hypotheses were mentioned in the Method. | The hypotheses were added to the Method.  Page 9, line 172 – 179  “Based on the available literature, the following hypotheses have been derived concerning cycling experience and the effectiveness of the balance bike training intervention: Hypothesis 1: It is anticipated that individuals with prior cycling experience, referred to as cyclists, will display superior performance in riding and controlling a balance bike compared to individuals without prior cycling experience, referred to as non-cyclists. Hypothesis 2: Non-cyclists who undergo the balance bike training intervention will demonstrate enhanced performance in balance bike tests and experience an increase in confidence levels when riding a two-wheel bike after engaging in practice with a balance bike.” |
